# Effectiveness of the Brazilian Visceral Leishmaniasis Surveillance and Control Programme in reducing the prevalence and incidence of *Leishmania infantum* infection

**DOI:** 10.1186/s13071-018-3166-0

**Published:** 2018-11-12

**Authors:** Iara Caixeta Marques da Rocha, Letícia Helena Marques dos Santos, Wendel Coura-Vital, Gisele Macedo Rodrigues da Cunha, Fernanda do Carmo Magalhães, Thais Almeida Marques da Silva, Maria Helena Franco Morais, Edward Oliveira, Ilka Afonso Reis, Mariângela Carneiro

**Affiliations:** 1 Laboratório de Epidemiologia das Doenças Infecciosas e Parasitárias, Departamento de Parasitologia, Instituto de Ciências Biológicas, Universidade Federal de Minas Gerais, Belo Horizonte, Minas Gerais Brazil; 2 Secretaria Municipal de Saúde de Belo Horizonte, Belo Horizonte, Minas Gerais Brazil; 3 Departamento de Análises Clínicas, Pós-graduação em Ciências Farmacêuticas, Escola de Farmácia, Universidade Federal de Ouro Preto, Ouro Preto, Minas Gerais Brazil; 4 Pós-graduação em Ciências Biológicas, Núcleo de Pesquisa em Ciências Biológicas, Universidade Federal de Ouro Preto, Ouro Preto, Minas Gerais Brazil; 5 Instituto de Ensino e Pesquisa da Santa Casa Belo Horizonte, Belo Horizonte, Minas Gerais Brazil; 6 Laboratório de Pesquisas Clínicas, Instituto René Rachou, Fundação Oswaldo Cruz, Belo Horizonte, Minas Gerais Brazil; 7 Departamento de Estatística, Instituto de Ciências Exatas, Universidade Federal de Minas Gerais, Belo Horizonte, Minas Gerais Brazil; 8 Pós-graduação em Ciências da Saúde: Infectologia e Medicina Tropical, Faculdade de Medicina, Universidade Federal de Minas Gerais, Belo Horizonte, Minas Gerais Brazil

**Keywords:** Control programme, Visceral leishmaniasis, Effectiveness, *Leishmania infantum* infection, Quasi-experimental study

## Abstract

**Background:**

Control strategies adopted by the Brazilian Visceral Leishmaniasis Surveillance and Control Programme (VLSCP) include identifying and culling seropositive infected dogs, early diagnosis and treatment of human cases, chemical control of the vector and population awareness. This study evaluated the effectiveness of the VLSCP on the prevalence and incidence rates of *Leishmania infantum* in children residing in areas under different VLSCP intervention times.

**Methods:**

A quasi-experimental epidemiological study with a panel (two cross-sectional) and a concurrent cohort was performed in three areas of Belo Horizonte, southeast Brazil. The first cross-sectional study (I) was carried out with 1875 children, 478 of which were enrolled in the cohort study. In the second cross-sectional study (II), 413 additional children were included, totalizing 891 children. Laboratory diagnosis was performed by ELISA-rK39. Analyses included multilevel logistic and Poisson regression models.

**Results:**

The incidence rates of *L. infantum* infection were: 14.4% in the area where VLSCP intervention was initiated in 2006 (AI2006); 21.1% in the area where intervention was initiated in 2008 (AI2008); and 11.6% in the area where intervention was initiated in 2010 (AI2010 - control area). A follow-up period of 24 months showed that the persons-time incidence rates in AI2006, AI2008, and AI2010 were: 6.2/100, 10/100, and 5.6/100 persons/24 months, respectively. The final prevalence rates of infection (cross-sectional II - in 2012), compared to the initial rates (cross-sectional I - in 2010), increased 83.7% in AI2006, 74.1% in AI2008, and decreased 5% in AI2010. Analysis of the effectiveness revealed that children residing in AI2008 are more likely to be infected (OR = 1.84; 95% CI: 1.06-3.23) and present a higher risk of infection (IRR = 1.76; 95% CI: 1.05-2.95) compared to those in AI2010. No statistically significant differences were observed in asymptomatic infection (OR and IRR) in AI2006 compared to AI2010.

**Conclusions:**

The VLSCP was not effective at controlling *L. infantum* infection in areas where interventions had respectively been carried out for six and four years. However, it is unclear what the consequences in terms of human infection and diseases would be in the absence of the VLSCP. Efforts to improve the effectiveness of control measures remain a necessary priority.

**Electronic supplementary material:**

The online version of this article (10.1186/s13071-018-3166-0) contains supplementary material, which is available to authorized users.

## Background

Visceral leishmaniasis (VL) is a neglected tropical disease that accounts for approximately 200,000 to 400,000 new cases annually worldwide [1, 2]. In the Americas, the etiological agent is the protozoan *Leishmania* (*Leishmania*) *infantum*, which is transmitted through the bite of the phlebotomine *Lutzomyia longipalpis*, with dogs being its main urban reservoir [3].

In the Americas, VL is present in 12 countries, with 90% of the cases being reported in Brazil (4200–6300 cases per year) and with a fatality rate around 7% [2, 4]. VL urbanization has been documented in Brazil since the 1980s and this trend represents a challenge for control measures in urban areas [5, 6]. The introduction and dispersion of *L. infantum* in urban areas is associated with a complex inter-dependent network and multiple factors such as environmental changes, migration, disorganized population growth and occupation of peripheral urban areas, high urban population density (both human and canine), and inadequate living conditions [4, 6, 7].

The Brazilian Visceral Leishmaniasis Surveillance and Control Programme (VLSCP) strategies include canine serological analysis followed by euthanasia of seropositive dogs, chemical control of the vector, early diagnosis and treatment of human cases, and population awareness [5]. In spite of the implementation of the VLSCP in endemic areas of Brazil, the control interventions have not been successful in interrupting transmission, especially in urban areas [8, 9]. In a systematic review analyzing intervention studies on the effectiveness of VL control programmes that included strategies to reduce the risk of transmission, such as animal reservoir control, vector control with insecticide spraying, or a combination of these interventions, Romero & Boelart [4] concluded that there is a lack of scientific evidence to sustain the effectiveness of these interventions in interrupting the spread of the disease. A randomized community intervention trial evaluating the impact of insecticide spraying and elimination of infected dogs on the incidence of human *L. infantum* infection showed that only dog culling reduced the human incidence, with estimates of effectiveness varying between 27–52%, depending on the analysis performed [10].

The first case of VL in Belo Horizonte, the capital of the state of Minas Gerais, located in southeastern Brazil, was confirmed in 1994 [11]. Since then, there has been significant investment in control measures implemented by the VLSCP [12, 13]. Between 1994 and 2016, 1762 cases were confirmed in Belo Horizonte with the lowest incidence rate of 1.2 cases per 100,000 inhabitants in 1998 and the highest rate of 7.2 cases per 100,000 inhabitants in 2008 [14].

Epidemiological studies in endemic areas highlight a large number of individuals infected by *L. infantum* with no clinical manifestations of the disease [15–20]. Indeed, infection by *L. infantum* affects a significant proportion of the population regardless of the occurrence of cases of the disease in one area [21]. Estimations of prevalence and incidence rates of asymptomatic infection in children appear to reflect recent parasite circulation given that they can indicate transmission cases in an endemic area [5]. Thus, these rates may be used as indicators for evaluation of the effectiveness of the strategies undertaken by the VLSCP in endemic areas [20, 22].

To understand the impact of the VLSCP in reducing the incidence of human VL, it is important to perform effectiveness evaluation under real conditions, i.e. under the routine of implementation and execution of the programme. The aim of the present study was to evaluate the effectiveness of the VLSCP on the prevalence and incidence rates of *L. infantum* infection in children less than ten years of age living in Belo Horizonte. We compared the areas where interventions had been carried out for six (intervention started in 2006) and four years (intervention started in 2008) with an area where the intervention of the VLSCP was being initiated (control area, intervention started in 2010).

## Methods

### Study area

Located in southeastern Brazil, Belo Horizonte is the capital city of the Brazilian State of Minas Gerais. It has 2,375,151 inhabitants and a population density of 7.2 inhabitants/km^2^. It is the sixth most populous city in Brazil, according to the census of the Brazilian Institute of Geography and Statistics [23]. The city is located at 852 m above sea level, 19°49'01"S, 43°57'21"W. The city has a dry winter and a hot and rainy summer, with an average annual temperature of 21 °C, average relative air humidity of 65%, and average annual rainfall of 1500 mm [23].

The study was conducted in three non-randomly selected geographically adjacent areas of Belo Horizonte. These three areas were selected based on the year that the VLSCP actions were initiated. The start date of implementation of the control strategies in each area was defined according to the guidelines and the strategic planning of the municipal public health authority, based on the epidemiological data of each area. In this assessment, the cumulative incidence of human visceral leishmaniasis (HVL) cases, as well as the social and environmental indicators were considered. A description of each area selected to take part in this study is presented below.

#### Intervention area 2006 (AI 2006)

This is the area where the VLSCP control strategies have been active the longest, as they started in 2006. The area has 20,672 inhabitants [24]. From 2006 to 2012, 13 cases of HVL were notified to the Brazilian Notifiable Disease Information System (Sistema de Informação de Agravos de Notificação, SINAN), and the cumulative incidence was 62.8/100,000 inhabitants [25]. The canine seroprevalence was 9.9% in the first year of intervention and 3.4% in 2012, the last year considered in this study. Vector chemical control was carried out in priority zones throughout all years of the study (2006–2012) [26].

#### Intervention area 2008 (AI 2008)

The VLSCP started in this area in 2008, which was considered an intermediate intervention time. The area has 22,591 inhabitants [24]. From 2006 to 2012, 11 HVL cases were notified to the SINAN, and the cumulative incidence in the area was 48.7/100,000 inhabitants [25]. The canine seroprevalence decreased from 12.7% in 2008 to 4.1% in 2012. Vector chemical control was carried out in priority zones in 2009, 2010 and 2012 according to strategic planning and epidemiological assessment [26].

#### Intervention area 2010 (AI 2010)

This area was selected as the control of the present study because the VLSCP was implemented only in 2010. The area has 16,818 inhabitants [24]. From 2006 to 2012, only one case of HVL was reported to the SINAN (in 2008), and the cumulative incidence was 5.9 cases/100,000 inhabitants [25]. In 2010, when the VLSCP started, the canine seroprevalence was 8.6% and decreased to 3.3% in 2012. No chemical vector control was used by the VLSCP until 2012 in this area [26].

### Study design

The investigation reported herein involves a quasi-experimental study encompassing a panel study (two cross-sectional) and a concurrent cohort study. The first cross-sectional study (I) established the baseline for the cohort, and the second (II) was carried out at the end of the investigation. Two indicators were estimated for the assessment of the effectiveness of the VLSCP using rK-39 antigen testing (ELISA-rK39): the incidence rate of infection and the difference between the final and the initial prevalence rates of asymptomatic infection. The study was conducted with children since infection in young individuals is an indicator of recent transmission. The inclusion criteria for the cross-sectional study I (baseline) was children aged 2–84 months. The exclusion criterion was applied only for the cohort study, which excluded the seropositive children from the follow-up.

#### Panel study: Cross-sectional study I

Conducted in 2010, this study established the baseline for the cohort study. It included 1875 children between 2 months and 7 years of age residing in all three areas (AI2006, AI2008, and AI2010). The study estimated the initial seroprevalence of asymptomatic *L. infantum* infection through ELISA-rK39.

#### Panel study: Cross-sectional study II

Conducted in 2012, this study involved 891 children between 2 months and 10 years of age residing in the three selected areas. At this stage, the final asymptomatic *L. infantum* infection seroprevalence rate was also estimated by ELISA-rK39.

#### Cohort study

**Fig. 1:**
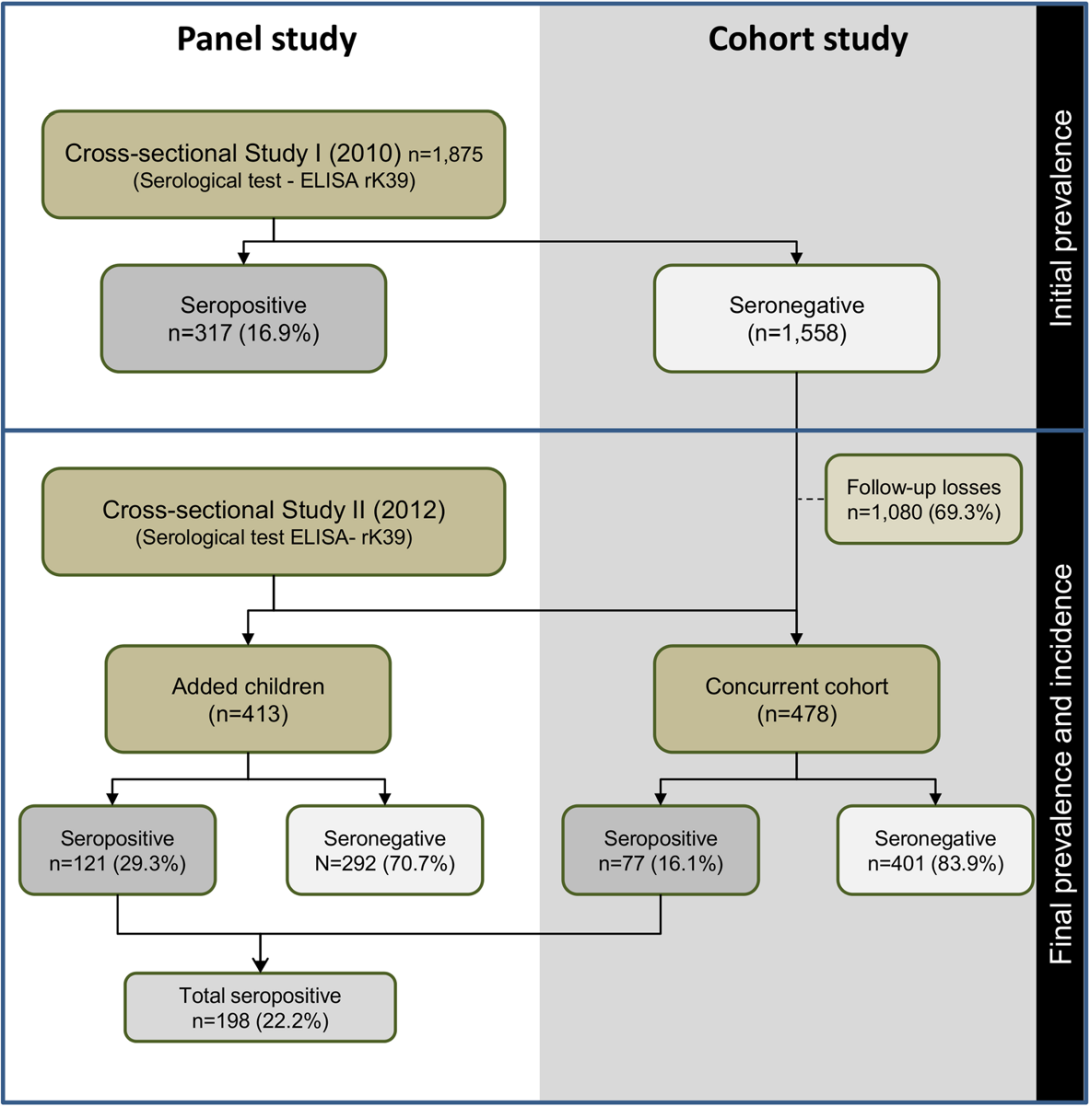
Study design: panel study (cross-sectional studies I and II) and cohort study. Belo Horizonte, Minas Gerais, Brazil

A total of 478 children diagnosed as seronegative for *L. infantum* infection during the first cross-sectional study (2010) were followed-up in the concurrent cohort study. This allowed us to estimate, using ELISA-rK39, the incidence rate of infection by *L. infantum* in 2012 (Fig. 1).

### Sampling

#### Panel Study: Cross-sectional study I (2010)

The sample size estimated for the study (baseline) was established using the following parameters: (i) an incidence among children between 9 months and 6 years of age of 2.7/100 children-years; (ii) type I error probability (α) equivalent to 0.05; (iii) a power for the statistical tests of (1-β) = 0.80; and (iv) a minimum 30% difference to be detected between the areas. Under these conditions, the sample size estimated for each area was 670 children of this age interval. In total, 1875 children residing in all three areas were assessed during the study.

#### Panel study: Cross-sectional study II (2012)

The sample size was calculated considering the following parameters: (i) the average prevalence rate of human infection of 15/100 children under 7 years of age, obtained from a previous study conducted in Belo Horizonte (19); (ii) a type I error probability (α) equivalent to 0.05; (iii) estimated precision of 0.05; and (iv) effect of design of 1.5. The sample size was calculated to be composed of approximately 300 children from each area, totalizing 900 children.

#### Cohort study

The cohort study was designed to follow up all the seronegative children identified in the cross-sectional study I. However, due to the loss of follow-up, it was conducted with all the seronegative children reached during the data collection.

### Data and biological sample collection, and serological testing

Throughout the study, the same procedures were used to collect data and blood samples. Initially, the children living in the study area were randomly selected based on the municipal census conducted through the Family Health Programme, a strategy currently adopted by the Brazilian Unified Health System (Sistema Único de Saúde, SUS). These data are periodically updated by community health workers who conduct a census in the areas studied.

Once the children were randomly selected, their parents and/or guardians were contacted by telephone or visited by someone from the team to explain the aims of the study. The data collection was conducted in 2012. The parents or legal guardians that agreed to take part in the study were visited by a team of nurse technicians who collected children’s blood samples and performed an interview. Before data collection, a free and informed consent form was signed by each legal guardian. Literate children were also asked to read and sign the informed consent form (adapted for children), thereby agreeing to the terms of the study. The blood was obtained by finger puncture and then transferred to filter paper, according to the protocol described by dos Santos et al. [20].

The ELISA rK-39 tests were carried out using serum obtained from the filter papers, following previously described procedures [21, 27]. The evaluations of rK-39 antigen performance for diagnosis of asymptomatic infection and of the disease showed that the sensitivity ranged between 70–92% and specificity between 77–100% [27–29].

The ELISA-rK39 seropositive children were invited to attend the public health unit for a clinical evaluation. On this occasion, blood samples were collected by venipuncture for complementary laboratory tests. These children were also clinically evaluated for signs and symptoms of VL.

A trained interviewer conducted the interview with the parents or legal guardians using a previously tested, structured questionnaire that sought information regarding the following groups of variables for: children (age, gender, behaviour); household characteristics (number of rooms, number of occupants and intra-domicile characteristics); peri-domicile environment characteristics (presence of backyard, presence and method of garbage disposal, i.e. collected, burned or buried, residual water destination, presence of domestic animals, presence of rubble and manure), knowledge about VL and its vector, and neighbourhood characteristics (presence of animals, trees, backyards and wastelands). Furthermore, we estimated the family income and social class according to the Brazilian socioeconomic classification criteria (Critério de Classificação Socioeconômica do Brasil, CCEB), which takes into account the level of education of the head of the family and the presence of specific domestic goods in the residence [30].

#### Assessment of the effectiveness of the Visceral Leishmaniasis Surveillance and Control Programme (VLSCP)

The questionnaire data and serological test results were codified and electronically stored in duplicate. The files were then compared and the divergences corrected. The software EpiData version 3.2 (Epidata Association, Odense Denmark, Europe) was used for data entry, and STATA version 12 (Stata Corp., College Station, TX, USA) was used for statistical analyses.

Infection prevalence rates were calculated for each area as a ratio between the number of seropositive children identified by the ELISA-rK39 test and the total number of children analyzed in the area. The percentage prevalence rate change was calculated by comparing the final rate (2012) against the initial rate (2010), according to the following equation:

$$ \mathrm{Change}\ \left(\%\right)=\frac{\left(\mathrm{Final}\ \mathrm{Prevalence}-\mathrm{Initial}\ \mathrm{Prevalence}\right)\times 100}{\mathrm{Initial}\ \mathrm{Prevalence}} $$

Incidence rates were calculated as the ratio between the numbers of new *L. infantum* seropositive cases, detected by ELISA-rK39, over the total number of children evaluated in the cohort, by area. The person-time incidence rate was estimated as the ratio between the number of new *L. infantum* infection seropositive cases over person-time in the follow-up period (24 months), per area evaluated. The denominator presented the follow-up time of the children evaluated in two time-points (2010 and 2012) plus half of the follow-up time of the children who were lost to the follow-up.

To evaluate the effectiveness of the VLSCP control strategies on the prevalence and incidence of seropositivity by *L. infantum* infection, two statistical models were used: multilevel logistic regression and Poisson regression with estimated robust variance. The multilevel logistic regression model was used in the cross-sectional studies to evaluate the impact of the intervention strategies adopted by the VLSCP on the final seroprevalence of asymptomatic infection in the studied areas using odds ratio (OR) with a 95% confidence interval (CI). At one level, this model considered the houses and, at a second level, the children’s characteristics. The residence was considered at the first level as, in some cases, more than one child from the same household were included in the study and were likely to share similar attributes due to the environment that is common to them. The Poisson regression model with robust variance estimation was used to evaluate the effectiveness of VLSCP intervention strategies on the incidence of new asymptomatic *L. infantum* infection cases in the cohort using the incidence rate ratio (IRR) with 95% CI.

Initially, bivariate analysis was performed in both models. Variables that showed a statistical association (*P* < 0.25) in the bivariate analysis were selected to compose the multivariate models according to the following groups: individual personal variables, socioeconomic, intra- and peri-domicile features, neighborhood, and knowledge of the vector population and the reservoir. Significant variables in all groups (*P* < 0.15) were selected for the multilevel logistic multivariate and Poisson models. Variables with more than two categories were converted into dummy variables. The building of the final models started with the full models, containing all variables, followed by successive discarding (backward selection) of the non-significant variables. The final multilevel logistic model to evaluate effectiveness was adjusted for variables that remained statistically significant (*P* < 0.05), thereby considering the effects of these variables on the prevalence of infection. The multilevel logistic regression was performed using STATA 12’s “xtmelogit” function. The final Poisson model with robust variance estimation to evaluate effectiveness was also adjusted for variables that remained significant (*P* < 0.05). This model considered the effects of these variables on the incidence of infection. The Poisson model was carried out using the “vcepoisson (robust) irr” function in STATA version 12.

## Results

### Study population

To establish the baseline for the study, 1875 children aged 2–84 months (mean: 42.1 months ± 24.7) were evaluated in 2010. Of these, 1558 were seronegative for *L. infantum*. Among those, 478 (31.4%) children were followed in the cohort and assessed again in 2012. The sample analyzed in the cohort study was composed of 160 children from AI2006, 180 children from AI2008 and 138 children from AI2010.

Among the seronegative children, 1080 were lost during the follow-up time. The percentages of losses were similar across all three areas: 71.8% in AI2006, 68.3 % in AI2008, and 67.3 % in AI2010. To characterize this loss, the variables age, gender, occupation and educational level of the household head, household income, presence of dogs, dogs with VL, and property features were compared between those followed and those who were lost during the follow-up. The greatest loss occurred in families with the lower incomes (*P* < 0.001) and lower level of education of the household head (*P* = 0.038).

The sample for the cross-sectional study II was initially composed of the 478 children from the follow-up cases. They were later joined by an additional 413 children, randomly selected, who did not participate in the baseline study, resulting in a sample size of 891 children aged 2–129 months (mean: 62 months ± 27.92). This sample was composed of 287 children from AI2006, 316 children from AI2008 and 288 children from AI2010.

Demographic characteristics of the children evaluated in the cohort and cross-sectional study II according to their area of residence are shown in Table 1. The age distributions of the children followed in the cohort study were statistically different among the areas: AI2010 had the highest proportion of children younger than 72 months (50.7%) and AI2006 and AI2008 had the highest proportion of children > 72 and ≤ 96 months (40.2 and 42.2%, respectively). There was no gender imbalance among the cohort children (Table 1). Considering the cross-sectional study II, the children evaluated presented similar age and gender in the three areas evaluated (Table 1).

**Table 1:** Characteristics of the children living in the three different areas evaluated, Belo Horizonte, Minas Gerais, Brazil

Study	Variable	AI 2006	AI 2008	AI 2010	*P*-value
		*n* (%)	*n* (%)	*n* (%)	
Cohort	Number of children	160 (33.5)	180 (37.7)	138 (28.9)	
	Age				
	≤ 48 months	13 (8.2)	17 (9.4)	28 (20.3)	0.03
	> 48 and ≤ 72 months	52 (32.7)	61 (33.9)	42 (30.4)	
	> 72 and ≤ 96 months	64 (40.2)	76 (42.2)	46 (33.3)	
	> 96 months	30 (18.9)	26 (14.5)	22 (16.0)	
	Gender				
	Male	78 (48.8)	91 (50.6)	77 (55.8)	0.46
	Female	82 (51.3)	89 (49.4)	61 (44.2)	
Cross-sectional-II	Number of children	287 (32.2)	316 (35.5)	288 (32.3)	
	Age				
	≤ 48 months	84 (29.3)	100 (31.6)	102 (35.4)	0.70
	> 48 and ≤ 72 months	77 (26.8)	91 (28.8)	75 (26.0)	
	> 72 and ≤ 96 months	93 (32.4)	96 (30.4)	81 (28.2)	
	> 96 months	33 (11.5)	29 (9.2)	30 (10.4)	
	Gender				
	Male	137 (47.7)	159 (50.3)	152 (52.8)	0.48
	Female	150 (52.3)	157 (49.7)	136 (47.2)	

*Abbreviations*: AI2006, area of intervention since 2006; AI2008, area of intervention since 2008; AI2010, area of intervention since 2010; CI, confidence interval

### Prevalence and incidence rates

In cross-sectional study I, the prevalence rates of asymptomatic *L. infantum* infection detected by ELISA-rK39 were 12.9% (87/675), 14.7% (100/679) and 17.9% (93/521) in AI2006, AI2008 and AI2010, respectively. The prevalence rates in cross-sectional study II were 23.7% (AI2006), 25.6% (AI2008) and 17.0% (AI2010). The relative change percentages showed an increase of 83.7% in AI2006 and 74.1% in AI2008, and a decrease of 5.3% in AI2010 (Table 2).

**Table 2:** Prevalence and incidence of *L. infantum* infection rates in areas with different intervention times by the Brazilian Visceral Leishmaniasis Surveillance and Control Programme, Belo Horizonte, Minas Gerais, Brazil

Areas	Prevalence 2010 (95% CI)	Prevalence 2012 (95% CI)	Change (%)	Incidence 2012 (95% CI)	Person-time incidence^a^ (95% CI)
AI2006	12.9 (10.6–15.6)	23.7 (19.1–28.9)	83.7	14.4 (9.8–20.7)	6.2 (4.0–9.1)
AI 2008	14.7 (12.3–17.6)	25.6 (21.1–30.7)	74.1	21.1 (15.8–27.6)	10.0 (7.2–13.6)
AI 2010	17.9 (14.8–21.4)	17.0 (13.1–21.8)	-5.3	11.6 (7.3–18.0)	5.6 (3.3–9.0)

^a^Denominator: Loss/2 + number of children on follow-up for 24 months for each area analyzed. Incidence rate/100 persons-24 months

*Abbreviations*: AI2006, area of intervention since 2006; AI2008, area of intervention since 2008; AI2010, area of intervention since 2010; CI, confidence interval

AI2006 had an incidence ratio (IR) of 14.4% and the person-time incidence rate of 6.2/100 persons/24 months. AI2008 had the highest incidence (IR = 21.1%) and person-time incidence rate of 10/100 persons/24 months. The control area (AI2010) presented the lowest IR (11.6%) and person-time incidence rate (5.6/100 persons/24 months) (Table 2).

### Characteristics of the three areas evaluated in the cohort study

In the cohort study, 398 households were visited and information from 478 children was analyzed. Table 3 shows the comparison of the families and household characteristics among the three areas evaluated in the cohort study. In all areas analyzed, it was observed that most household heads had completed high school. AI2010 showed the largest number of household heads with higher education, either in the course or completed (23.9%), whereas AI2006 showed the highest percentage of illiterate individuals (12%). According to the CCEB [30], most of the children living in AI2006 (65.5%) and AI2008 (58.7%) belonged to socioeconomic class C or below, with a monthly income below 2.4 of minimum monthly wage (BMMW; in 2012, the Brazilian minimum monthly wage was equivalent to 311 USD). Children from AI2010 belonged mainly (58.4%) to class B2 or lower (monthly income ≤ 4.1 BMMW). Over half of the visited residences in all three areas had dogs, plants in the peri-domicile, and trees in the neighborhood. The presence of chickens was more frequently observed in AI2006 (14.1%) and AI2008 (12.6%), which were the areas with the highest percentage of families from lower socioeconomic classes. Statistically significant differences were found among all three areas concerning “educational level of the head of the family” and “families’ socioeconomic class” (Table 3).

**Table 3:** Comparison of the families and household characteristics among the three areas evaluated in the cohort study, Belo Horizonte, Minas Gerais, Brazil

Variable	AI 2006	AI 2008	AI 2010	*P*-value
	*n* (%)	*n* (%)	*n* (%)	
Number of residences	142 (35.7)	143 (35.9)	113 (28.4)	
Education level of the household head				
Higher education (in course or completed)	15 (10. 6)	7 (4.9)	27 (23.9)	0.001
High-school (completed)	60 (42.3)	77 (53.9)	51 (45.1)	
Foundation (completed)	28 (19.7)	26 (18.2)	16 (14.2)	
Primary school (completed)	22 (15.5)	30 (21.0)	13 (11.5)	
Iliterate	17 (11.9)	3 (2.10)	6 (5.31)	
Socio-economic class (BMMW)				
A1, A2 and B1 (20.8 to 4.2)	16 (11.3)	18 (12.6)	30 (26.6)	0.001
B2 (4.1 to 2.5)	33 (23.2)	41 (28.7)	44 (38.9)	
C1, C2 and D (2.4 to 1.1)	93 (65.5)	84 (58.7)	39 (34.5)	
Chickens				
Present	20 (14.1)	18 (12.6)	10 (8.9)	0.43
Absent	122 (85.9)	125 (87.4)	103 (91.2)	
Dogs				
Present	75 (52.8)	80 (55.9)	60 (53.1)	0.85
Absent	67 (47.2)	63 (44.1)	53 (46.9)	
Peridomiciliar plants				
Present	107 (75.4)	98 (68.5)	87 (77.0)	0.15
Absent	33 (23.24)	45 (31.47)	26 (23.0)	
Neighbourhood trees				
Present	92 (64.8)	89 (62.2)	75 (66.4)	0.78
Absent	50 (35.2)	54 (37.8)	38 (33.6)	
Rubble				
Present	55 (38.7)	39 (27.3)	29 (25.7)	0.06
Absent	85 (59.9)	101 (70.6)	84 (74.3)	
Gully				
Present	46 (32.4)	28 (19.6)	24 (21.2)	0.07
Absent	95 (66.9)	115 (80.4)	88 (77.9)	
Waste				
Present	18 (12.7)	18 (12.6)	21 (18.6)	0.21
Absent	122 (85.9)	125 (87.4)	92 (81.4)	

*Abbreviations*: AI2006, area of intervention since 2006; AI2008, area of intervention since 2008; AI2010, area of intervention since 2010; BMMW, Brazilian monthly minimum wage; Class A1, A2 and B1 (20.8 to 4.2 BMMW); Class B2, (4.1 to 2.5 BMMW); Class C1, C2 and D (2.4 to 1.1 BMMW)

### Effectiveness of the control strategies adopted by the VLSCP on reducing *L. infantum* incidence rates

The final Poisson model used to evaluate the effectiveness of the control strategies adopted by the VLSCP on reducing *L. infantum* incidence rates showed that children living in AI2008 presented higher IRR (1.82, *P* = 0.03) than those residing in the control area AI2010 when adjusted according to the child’s age, family economic class, and the presence of trees in the neighborhood. In AI2006, the VLSCP interventions showed no statistically significant difference in the IRR of asymptomatic *L. infantum* infection (1.24, *P* = 0.48) compared with AI2010 when adjusted to the child’s age, family economic class, and the presence of trees in the neighborhood (Table 4).

**Table 4:** Effectiveness of the control strategies adopted by the Brazilian Visceral Leishmaniasis Surveillance and Control Programme, Belo Horizonte, Minas Gerais, Brazil

Intervention área	IRR^a^	95% CI	*P*-value	IRR^b^	*P*-value	95% CI
AI2010	1					
AI2008	1.82	1.06–3.13	0.03	1.76	0.03	1.05–2.95
AI2006	1.24	0.68–2.25	0.48	1.18	0.59	0.65–2.14

^a^Non-adjusted model

^b^Model adjusted to age of the child, family’s socio-economic class and presence of trees in the neighborhood

*Abbreviations*: AI2006, area of intervention since 2006; AI2008, area of intervention since 2008; AI2010, area of intervention since 2010; CI, confidence interval; IRR, incidence rate ratio

### Characteristics of the three areas evaluated in cross-sectional study II

The characteristics of the population evaluated in the second cross-sectional study were very similar to those of the population in the cohort. In cross-sectional study II, 659 households were visited and 891 children participated. Table 5 shows the comparisons of selected characteristics of the families and households among the three areas evaluated in cross-sectional study II. In all areas analyzed, high school was the educational level most frequently completed by the household head. The maximum percentage of household heads with higher education in course or complete was observed in AI2010 (21.8%), whereas AI2006 presented the highest percentage of illiterate individuals (9.2%).

**Table 5:** Comparison of the families and household characteristics among the three areas evaluated in the cross-sectional study, Belo Horizonte, Minas Gerais, Brazil

Variables	AI 2006	AI 2008	AI 2010	*P*-value
	*n* (%)	*n* (%)	*n* (%)	
Number of households	229	223	207	
Education level of the household head				
Higher education in course or completed	21 (9.2)	16 (7.2)	45 (21.8)	0.001
High-school (completed)	103 (45.2)	121 (54.3)	86 (41.7)	
Foundation (completed)	51 (22.4)	40 (17.9)	36 (17.5)	
Primary school (completed)	32 (14.0)	41 (18.4)	27 (13.1)	
Illiterate	21 (9.2)	5 (2.2)	12 (5.8)	
Socio-economic class (BMMW)				
A1, A2 and B1 (20.8 to 4.2)	24 (10.5)	31 (13.9)	49 (23. 7)	0.001
B2 (4.1 to 2.5)	54 (23.6)	66 (29.6)	72 (34.8)	
C1, C2 and D (2.4 to 1.1)	151 (65.9)	126 (56.5)	86 (41.5)	
Chickens				
Present	34 (14.8)	24 (10.8)	18 (8.7)	0.12
Absent	195 (85.1)	199 (89.2)	189 (91.3)	
Dogs				
Present	119 (51.9)	120 (53.8)	104 (50.2)	0.76
Absent	110 (48.0)	103 (46.2)	103 (49.8)	
Peridomicilar plants				
Present	162 (70.7)	145 (65.0)	151 (72.9)	0.02
Absent	65 (28.4)	78 (35.0)	55 (26.6)	
Neighbourhood trees				
Present	148 (64.6)	139 (62.3)	136 (65.7)	0.75
Absent	81 (35.4)	84 (37.7)	71 (34.3)	
Rubble				
Present	93 (40.6)	68 (30.5)	56 (27.1)	0.01
Absent	132 (57.6)	153 (68.6)	151(72.9)	
Gully				
Present	82 (35.8)	49 (22.0)	42 (20.3)	0.001
Absent	144 (62.9)	174 (78.0)	164 (79.2)	
Waste				
Present	35 (15.3)	30 (13.5)	31 (15.0)	0.19
Absent	191(83.4)	193 (86.5)	176 (85.0)	

*Abbreviations*: AI2006, area of intervention since 2006; AI2008, area of intervention since 2008; AI2010, area of intervention since 2010; BMMW, Brazilian monthly minimum wage; Class A1, A2 and B1 (20.8 to 4.2 BMMW); Class B2, (4.1 to 2.5 BMMW); Class C1, C2 and D (2.4 to 1.1 BMMW)

According to the CCEB [30], most of the children living in AI2006 (65.9%) and AI2008 (56.5%) belonged to the socioeconomic class C or lower (≤ 2.4 BMMW). On the other hand, children from AI2010 belonged mainly to the socioeconomic class B or higher (58.4%) (monthly income between 4.1 and 20.8 BMMW). The presence of dogs, plants and trees in the household neighborhood were characteristics commonly found in most households of all three areas (*n* = 659). The presence of chickens was more frequent in residences of AI2006 (14.8%) and AI2008 (10.8%), which were the ones with the highest rate of families belonging to the lower socioeconomic classes, according to CCEB [30]. Statistically significant differences were found in all three areas for the following variables: level of education of the household head (*P* < 0.01), family’s socioeconomic class (*P* < 0.01), the presence of rubble (*P* = 0.01), and the presence of a gully (*P* < 0.01) (Table 5).

### Effectiveness of control strategies on reducing the prevalence of asymptomatic *L. infantum* infection

The final multivariate multilevel logistic model used to evaluate the effectiveness of the VLSCP on reducing the prevalence of infection showed that children living in AI2008 were more likely to be infected (OR = 1.94; *P* = 0.02) than the children from the control area (AI2010) when adjusted for the child’s age, socioeconomic class, the presence of trees in the neighborhood and the presence of chickens (Table 6). The chance of children in AI2006 to be infected was not significantly different from those living in AI2010 (OR = 1.71; *P* = 0.07) when adjusted for the child’s age, socioeconomic class, the presence of trees in the neighborhood and the presence of chickens (Table 6).

**Table 6:** Effectiveness of the control strategies adopted by the Brazilian Visceral Leishmaniasis Surveillance and Control Programme in transversal study II, Belo Horizonte, Minas Gerais, Brazil

Intervention área	OR^a^	95% CI	*P*-value	OR^b^	*P*-value	95% CI
AI2010	1					
AI2008	1.94	1.1–3.42	0.02	1.84	0.03	1.06–3.23
AI2006	1.71	1.1–3.42	0.07	1.68	0.07	0.94–2.98

^a^Non-adjusted model

^b^Model adjusted by age of the child, socio-economic class of the family, presence of trees in the neighborhood and presence of chickens.

*Abbreviations*: AI2006, area of intervention since 2006; AI2008, area of intervention since 2008; AI2010, area of intervention since 2010; OR, odds ratio; CI, confidence interval

## Discussion

The present study showed that the VLSCP was not effective at reducing HVL transmission, as evaluated by the prevalence and incidence of asymptomatic infection by *L. infantum* in children living in areas where the control programme had respectively been carried out for six and four years, compared with the control area where the programme had just been initiated. The prevalence of infection with *L. infantum* showed an increase in AI2006 (83.7%) and AI2008 (74.1%), the two areas where the VLSCP had been previously implemented, and a slight reduction (5%) in the control area AI2010. The incidence and persons-years incidence rates after two years of follow-up were higher in AI2008 (21.1% and 10/100 persons-24 months), followed by AI2006 (14.4% and 6.2/100 persons-24 months) and AI2010 (11.6% and 5.6/100 persons-24 months).

It was expected that the areas AI2006 and AI2008 would present a decrease in the prevalence rates and lower incidence rates of asymptomatic infection compared with the control area (AI2010). Despite the increase of the prevalence rate in AI2006 when compared with the control area (AI2010), after adjustment for confounding factors in the Poisson regression model, the risk of infection (IRR = 1.18) was not statistically significant. AI2008 showed higher final prevalence and incidence rates, and a higher risk of infection (IRR = 1.76) when compared with the control area (AI2010). These results suggest that despite the intervention adopted by the VLSCP in the areas, high transmission occurred among children, especially in AI2008.

A small decrease (5%) was observed in the final prevalence rate in the control area (AI2010) compared with the initial prevalence. Additionally, this area showed the lowest incidence rates of infection compared with the other areas under VLSCP intervention. Despite the short intervention time, the control strategies implemented in this area in 2010 resulted in a small reduction in prevalence. It is likely that the higher socioeconomic level and the healthier environmental conditions and urbanization of this area better supported the control measures in comparison with the other areas of intervention. Despite the adjustments made to confounding variables in the statistical analysis models, one could infer that epidemiological and intra-urban characteristics in this area may be associated with the reduction in transmission. One ecological study [31] conducted in Belo Horizonte to identify the areas at greater risk for HVL infection and the risk factors involved in transmission corroborate our results. According to the authors, the relative risk of VL was shown to be correlated with lower income and educational level. Since AI2006 and AI2008 presented worse socioeconomic levels and schooling when compared to AI2010, we can expect that these factors may have interfered in the control actions. A point that deserves emphasis is that the baseline prevalence rates of *L. infantum* infection were comparable among the three areas, thus increasing the internal validity of this study, an important condition for programme evaluation studies [32].

A quasi-experimental design, which permits control of external factors or confounding variables without random allocation of the areas under intervention, has been the method of choice to evaluate the effectiveness of health programmes. A panel study (two cross-sectional studies) conducted in the same population in two-time intervals is the best model for demonstrating changes in the prevalence of infection [32], and here, unlike a conventional cross-sectional study, longitudinal measurements of samples of children population were obtained. Even with the limitations associated with losses in the cohort study, caused by the long period between the two evaluations (two years), the analysis of the cohort is of great relevance as it allows for the estimation of incidence, the most appropriate measure in the understanding of disease transmission. In the search for more reliable data, we evaluated incidence after two years of follow-up and a second prevalence with the inclusion of children in the study. This approach allowed us to assess the effectiveness of VLSCP on *L. infantum* infection through different analyses.

Studies that evaluated the strategies adopted by the VLSCP such as the culling of seropositive dogs and insecticide spraying have shown that these measures are not effective in interrupting the human transmission, especially in urban areas. In the study by Souza et al. [22], although the data suggested a small reduction in the incidence of human infection in areas of intervention when compared with the control area, the difference was not statistically significant. The study by Werneck et al. [10] compared different control strategies and showed the low effectiveness of the euthanasia of dogs on the incidence of human infection. On the other hand, a study by Costa et al. [33] pointed towards a protective effect of the euthanasia of infected dogs in the human incidence of *L. infantum* infection associated with intra-domicile spraying. However, spraying the peridomicile, associated or not with canine euthanasia, had no significant protective effect in human transmission. Dietze et al. [34] conducted an intervention study with the aim of determining the impact of the elimination of dogs in controlling the disease. Twelve months after the start of the trial, they observed an increase in HVL seroprevalence in intervention and control areas, whereas canine seroconversion declined in all areas.

The evaluation of the effectiveness of VL control strategies performed herein did not consider VLSCP’s indicators of the operational process, such as the number of households visited, the number of examined and euthanized dogs, the time between identification of seropositive dogs and euthanasia, and coverage of insecticide spraying. The assumption was that the programme had been implemented following pre-established strategies based on epidemiological criteria for each area, according to the recommendation of the VLSCP. This assumption can be supported by a study conducted to evaluate the VLSCP activities in Belo Horizonte from 2007 to 2011 [13], which showed a reduction in some indicators of the programme results, such as canine seroprevalence (47.8%) and the incidence of HVL cases (from 7.2 to 3.9/100,000 inhabitants). Additionally, the results showed that more than 85% of the seropositive dogs were euthanized, but only 57.5% of vector control coverage was reached in 2010. Although the authors concluded that the programme objectives were achieved in Belo Horizonte, revision of the proposed actions by the control programme was necessary due to the complexity of the interventions [13].

Problems related to the effectiveness of the VL programme have been pointed out by many authors and include the inaccuracy of the serological test to identify canine infection, the time between the identification of canine infection and euthanasia, substitution of sacrificed dogs by new dogs, absence of an official information system for recording and monitoring canine control activities, insufficient knowledge about the vector behavior, low coverage of insecticide spraying, and the lack of sustainability of the control strategies [9, 33, 35–37]. Although the VLSCP has clear objectives and strategies, it does not propose ways of evaluating its actions. A study conducted to evaluate the control activities demonstrated the importance of evaluating the set of control actions in their routine, through indicators that signal their positive and critical points [13].

The seropositive children identified in the present study were examined by a physician, and new blood samples were collected for other serological (ELISA-rk39 and ELISA with soluble antigen) and molecular (qPCR) tests. No seropositive children at follow-up developed any clinical signs or symptoms of VL and therefore were not treated since the treatment is indicated only for patients with clinical manifestations [5]. These results are in accordance with other studies conducted in Brazil, which clinically monitored asymptomatic individuals with positive results for ELISA-rK39 and revealed that none of the followed individuals developed the disease [18, 19, 38, 39].

A few limitations of the present study need to be mentioned. First, the difficulty in defining *L. infantum* infection cases due to the absence of an accurate diagnostic test for the detection of the asymptomatic infection. The drawback of the diagnostic test is due to the low levels of circulating antibodies in the serum of asymptomatic individuals as the parasitic load is scarce at this stage of the disease [20, 27, 38]. However, given that the same diagnostic procedures were carried out throughout the study, the misclassification bias is probably the same in the different moments and areas evaluated. Secondly, the loss of follow-up was high, and selection bias should be considered. However, the follow-up loss was homogeneous among the three areas, making the data comparable. Conversely, comparison of demographic and socioeconomic characteristics between the participants fully followed-up and those that were lost during the follow-up period revealed socioeconomic differences. The highest proportion of losses was found among participants of the lower socioeconomic classes, as well as among those whose household heads had a lower educational level. It is likely that these families do not own their homes and have greater mobility. In spite of this difference, selection bias may be minimized since the losses were similar among the areas. However, the incidence infection estimates were probably less precise due to the losses during follow-up. The mobility should have little impact on the effectiveness of the programme, as the control measures are implemented in a continuous way with broad actions and focusing on the collective health. Furthermore, in transversal study II, a difference in seroprevalence was observed between the children that were included and those followed in the cohort study. This may be due to age differences, as the children in the follow-up (mean 75 months and median 76 months) were older than the children who were included (mean 47 months and median 47 months). Younger children may be more susceptible to infection. To try to control the age effect, the final model of effectiveness was adjusted by the variable age.

In summary, the present study showed that the prevalence and incidence of *L. infantum* infection increased during the study period in areas under intervention, indicating that the VLSCP was not effective in reducing asymptomatic infection. In contrast, data from the municipal public health services showed that the human disease decreased over the study period in AI2006 and AI2008 (Additional file 1: Table S1). Regarding the areas evaluated, the incidence of VL cases reported to SINAN varied as follows: AI2006, six cases to one case (13 cases during the period); AI2008, two cases to one case (11 cases during the period); AI2010, only one case was reported in the period. Therefore, evaluating the impact of interventions on the incidence of HVL was impracticable due to the small number of clinical cases. It is important to note that indicators involving rare events may cause distortions in the understanding of the results. The importance of the asymptomatic infected individuals in the epidemiology of the disease remains unclear. We would like to point out that the findings must be interpreted with caution since the intervention period analyzed may be too short to draw a conclusive assessment of the effectiveness of the strategies implemented by the VLSCP. The asymptomatic infection in children is probably an indicator of parasite transmission in a given area and does not predict the occurrence of the human disease, as recently demonstrated in a study conducted in Belo Horizonte [21].

The advantages of our study include the evaluation of the effectiveness of the VLSCP without interrupting or interfering with the programme routine. Therefore, assessing the impact of control actions on reducing human infection becomes more real. when it was carried out concomitantly with the actions of the programme.

## Conclusions

Evaluation of the strategies adopted by the VLSCP have shown that the control interventions have not been successful in interrupting *L. infantum* transmission, especially in urban areas. The evaluation of effectiveness performed herein showed that the control measures adopted by the VLSCP did not significantly affect asymptomatic infection levels in three urban areas where the programme had been active for different lengths of time. Although the importance of asymptomatic individuals in areas of active transmission needs to be better investigated, it is unclear what the result on infection and illness would be if the VLSCP strategies were absent in the areas analyzed by the present study. There are gaps in the knowledge regarding the urbanization of VL that hamper the effective action of the control programme in Brazil. Therefore, efforts to optimize and improve the effectiveness of control measures remain a necessary priority.

## Additional file

Additional file 1: **Table S1.** Temporal series of the canine prevalence and human visceral leishmaniasis (HVL) cases in three areas with different intervention times by the Brazilian Visceral Leishmaniasis Surveillance and Control Programme (VLSCP), Belo Horizonte, Minas Gerais, Brazil. (DOC 46 kb)

## Abbreviations

AI: Area of intervention
CCEB: Critério de Classificação Socioeconômica do Brasil
CI: Confidence interval
HVL: Human visceral leishmaniasis
IR: Incidence rate
BMW: Brazilian minimum wage
OR: Odds ratio
RR: Incidence ratio rate
SINAN: Sistema de Informação de Agravos de Notificação
VL: Visceral leishmaniasis
VLSCP: Visceral Leishmaniasis Surveillance and Control Programme
