# Supplementary material for: Effectiveness of the Brazilian Visceral Leishmaniasis Surveillance and Control Programme in reducing the prevalence and incidence of Leishmania infantum infection
Source: Parasit Vectors. 2018 Nov 12;11:586. doi: 10.1186/s13071-018-3166-0 (PMC6233359; doi:10.1186/s13071-018-3166-0)
Supplement: Supplementary file 1 — Table S1. Temporal series of the canine prevalence and human visceral leishmaniasis (HVL) cases in three areas with different intervention times by the Brazilian Visceral Leishmaniasis Surveillance and Control Programme (VLSCP), Belo Horizonte, Minas Gerais, Brazil. (DOC 46 kb) [file 13071_2018_3166_MOESM1_ESM.doc]

**Additional file 1:**

**Table S1.** Temporal series of the canine prevalence and human visceral leishmaniasis (HVL) cases in three areas

with different intervention times by the Brazilian Visceral Leishmaniasis Surveillance and Control Programme,

Belo Horizonte, Minas Gerais, Brazil

|  | **AI2006** | | | | | **AI2008** | | | | | **AI2010** | | | | |
| --- | --- | --- | --- | --- | --- | --- | --- | --- | --- | --- | --- | --- | --- | --- | --- |
| **Year** |  | Collected samples | | Canine prevalence | HVL cases |  | Collected samples | Canine prevalence | | HVL cases |  | Collected samples | Canine prevalence | | HVL cases |
|  |  | N | N (%) | | N |  | N | | N (%) | N |  | N | | N (%) | N |
| **2006** |  | 2088 | 233 (11.2) | | 6 |  | ~~-~~ | | ~~-~~ | 2 |  | ~~-~~ | | ~~-~~ | 0 |
| **2007** |  | 3178 | 297 (9.3) | | 4 |  | ~~-~~ | | ~~-~~ | 2 |  | ~~-~~ | | ~~-~~ | 0 |
| **2008** |  | 2819 | 267 (9.5) | | 1 |  | 3480 | | 442 (12.7) | 2 |  | ~~-~~ | | ~~-~~ | 1 |
| **2009** |  | 2620 | 179 (6.8) | | 1 |  | 2948 | | 223 (7.5) | 1 |  | ~~-~~ | | ~~-~~ | 0 |
| **2010** |  | 2424 | 229 (9.4) | | 0 |  | 2886 | | 205 (7.0) | 1 |  | 1638 | | 145 (8.6) | 0 |
| **2011** |  | 2410 | 147 (6.1) | | 0 |  | 3044 | | 149 (4.9) | 1 |  | - | | - | 0 |
| **2012** |  | 2407 | 73 (3.0) | | 1 |  | 3122 | | 107 (3.4) | 1 |  | 1092 | | 36 (3.3) | 0 |

AI2006: Area of Intervention since 2006; AI2008: Area of Intervention since 2008; and AI2010: Area of Intervention since 2010.

Sources: Zoonosis Control System and Notifiable Diseases Information System (SINAN)
